# A routing method with adaptively adjusting memory information based on local routing history

**DOI:** 10.1371/journal.pone.0283472

**Published:** 2023-04-19

**Authors:** Takayuki Kimura, Yutaka Shimada

**Affiliations:** 1 Department of Electrical, Electronics and Communication Engineering, Faculty of Fundamental Engineering, Nippon Institute of Technology, Miyashiro, Saitama, Japan; 2 Department of Information and Computer Sciences, Graduate School of Sciences and Engineering, Saitama University, Saitama, Japan; Khon Kaen University, THAILAND

## Abstract

Finding the shortest paths for packets from sources to destinations in packet-switched communication networks is an inevitable problem in building a future high-speed information society. A routing method with memory information has already been proposed to alleviate the congestion of large volumes of packet flow. This routing method shows a high transmission completion rate even for large volumes of packet flows in communication networks with scale-free properties. However, the method exhibits poor performance for networks with local triangular connections and long distances between nodes. To overcome these problems, in this study, we first enhanced the routing performance of the conventional communication network models by using the betweenness centrality of nodes, which is one of the network centralities that measures the number of shortest paths that pass through each node in the networks. Subsequently, we adaptively changed the transmitting paths of packets by using only local information. Numerical simulations indicated that our routing method performs successfully for various topologies of communication networks by avoiding the congested node, and effectively using the memory information.

## Introduction

The recent significant development of communication technologies, such as the Internet of Things, various applications using cloud communication, and the fifth generation communication will result in a huge amount of traffic to the packet-switched communication networks. The shortest path routing (SP) method is commonly employed as a routing method in real communication networks. This method successfully handles the transmission of small volumes of traffic flows in the communication networks from their sources to destinations. However, for large traffic volumes, the transmission of data to their destinations is hindered because of existing scale-free (SF) properties in real communication networks [1]; the packets are easily congested at routers, where many transmitting paths go through. One of the solutions to avoid traffic congestion is to rewire the physical connections between any hosts or routers. However, this requires huge costs. The second solution is to implement a sophisticated routing method that effectively diversifies the transmitting routes for packets depending on the traffic volumes and the underlying topologies in the communication networks.

Considering the conventional routing methods that aim to address data transmission in high-traffic communication networks, Echenique *et al*. [2] proposed a routing method that employs distance information and the number of stored packets at nodes. Ling *et al*. [3] proposed a global dynamic routing that uses stored information of packets in the entire communication network. Tang *et al*. [4] proposed a self-adjusting traffic awareness protocol that utilizes the connectivity of communication networks and the stored information of packets. Wang *et al*. [5, 6] analyzed the traffic dynamics of communication networks with SF properties and proposed a routing algorithm that integrates the local static and dynamic information. Yang *et al*. [7, 8] proposed an effective routing method for mobile networks. Gao *et al*. [9] employed weights between nodes that dynamically change based on the connectivity of the network and stored information of packets. Echagüe *et al*. [10, 11] proposed a routing algorithm that dynamically changes the distance between nodes using congestion parameters based on the number of packets that arrive at the nodes. Lin *et al*. [12] proposed a routing strategy with a restrictive queue length algorithm, which reduces the load of the hub nodes in the networks. Zhang *et al*. [13] proposed the routing strategy in which each packet at a node has a different level of priority for the next transmitting node. Horiguchi *et al*. [14] proposed a routing strategy by using the Hopfield and Tank typed mutually connected neural networks, and this method was further improved by reinforcement of a learning strategy [15] and incorporation of the stochastic effect [16]. Recently, Hou *et al*. proposed QoS routing algorithms based on the particle swarm optimization [17] and the ant colony optimization [18] for different types of services in named data networking, which are the next generation of network architectures. The above-mentioned routing methods [2, 9–11, 14–16] require real-time information such as the number of stored packets at adjacent (or connected) nodes to determine the next transmitting node. However, such information cannot always and instantaneously be obtained in real networks. Therefore, a routing method that avoids packet congestion using only local information is required.

From this viewpoint, a routing method that autonomously diversifies packet transmissions using chaotic neurodynamics has been proposed [19, 20]. An advantage of using chaotic neurodynamics is the refractory effect, which inhibits the firing of neurons for a certain period of time [21]. By using this functionality, the solution methods with the chaotic neurodynamics were applied to various types of combinatorial optimization problems [22]. In the routing method using the chaotic neurodynamics, the refractory effect works to memorize the histories of packet transmissions at each node. Further, a routing method using memory information, which reinforces the packet memorizing functionality in chaotic neural networks, has already been proposed [23]. According to the hop distance information and memory information, this routing method [23] avoids the congestion of packets and maintains a high transmission completion rate of packets for the communication networks with SF properties. In addition, the effectiveness of the memory information is clarified by using the method of surrogate data [24], which is a statistical hypothesis testing used in the research field of nonlinear time-series analysis [25]. However, the routing method using memory information showed poor performance for networks with highly clustered and long distances between nodes [24].

In this work, we incorporated two strategies to improve the routing method using memory information [23, 24]. First, we reformed the conventional communication network models [26] by changing the number of transmissions of packets and buffer sizes at each node based on node betweenness centrality, which is one of the network centralities that measures the number of shortest paths that pass through nodes in the networks [27]. Second, we enhanced the performance of the routing method [24] by adaptively adjusting the weight of memory information at each node based on the transmitting probabilities. Numerical experiments indicated that our proposed routing method that adaptively adjusted the weights of the memory information worked well both for communication networks with SF properties and also highly clustered networks with long node distances.

## Communication network models

This study used communication network models represented by an unweighted and undirected graph *U* = (*V*, *E*), where *V* is a set of nodes, and *E* is a set of edges [2, 4, 9–11, 20, 24, 28, 29]. In the communication network model, each node represents a host and a router, and each edge represents a connection between the nodes. A packet is generated at a randomly selected node (source), and a randomly selected destination different from the source is assigned. Each node has a buffer for storing packets. If a packet is generated at a node, it is stored at the tail of the buffer of the node. A packet is removed from the network if it is transmitted to a node with a full volume of the packets in its buffer or in case the packet arrives at its destination. All packets are transmitted according to the first-in-first-out principle.

We first enhanced the routing performance of communication network models proposed in Ref. [26] by improving the number of transmissions of packets and buffer sizes at each node. To show that the conventional communication network models [23, 24, 26] have poor packet routing abilities, we first displayed relationships between degree and node betweenness centralities of networks and the number of existing packets by using the *shortest path* (SP) method. The SP method transmits packets from sources to destinations using uniquely determined shortest paths. In this work, if multiple adjacent nodes have the same shortest hop distance to the designations, we assigned an adjacent node that has the youngest index as the transmitting node of packets. The shortest paths for all pairs of nodes are preliminarily obtained once the networks were constructed using the shortest path algorithm, such as the Dijkstra algorithm [30].

In Ref [26], the storage performance of node *i* that corresponds to the buffer sizes at each node, *B*_*i*_, is defined by:

$$B_{i}=\beta k_{i},$$ (1)

where *β* > 0 is a control parameter and *k*_*i*_ is the degree of node *i*, which corresponds to the number of edges of a node. By using Eq (1), each node has space for storing packets, which is proportional to its degree. Next, the transmission performance of node *i* that corresponds to the number of transmissions of packet at each node, *C*_*i*_, is defined as:

$$\begin{array}{c} C_{i}=1+\left\lfloor \gamma k_{i}+0.5\right\rfloor , \end{array}$$ (2)

where *γ* > 0 is a control parameter and ⌊⋅⌋ expresses the floor function. By using Eq (2), the node that has a large degree transmits many packets to its adjacent (connected) nodes during one iteration. In this work, one iteration is defined as a period for each node to transmit the first *C*_*i*_, ∀*i* ∈ *V* packets to the adjacent nodes.

Next, we evaluated the SP method for the conventional and improved communication network models. In all the numerical experiments, we set the number of iterations, denoted by *I*, as *I* = 10^3^. In addition, we set *β* and *γ* to 10^3^ and 0.4, respectively, based on conventional studies [23, 24]. We also generated *R* packets at each iteration.

We generated SF networks, small-world networks, and networks with both scale-free and small-world properties by using the Barabási and Albert (BA) [1], the Watts and Strogatz (WS) [31], and the Klemm and Eguíluz (KE) [32] models, respectively. The BA model [1] was constructed using the following procedure. We started with a complete graph of *m* nodes, and inserted a new node with *m* edges at every time step. Next, we connected the *m* edges of the newly added node to the nodes that already existed in the network with probability $\Pi \left(k_{i}\right)=k_{i}^{\left(t\right)}/\sum_{m=1}^{|V^{\left(t\right)}|}k_{m}^{\left(t\right)}$, where $k_{i}^{\left(t\right)}$ is the degree of node *i* (*i* = 1, …, |*V*^(*t*)^|) at time *t*, *V*^(*t*)^ is a set of indices of pre-existing nodes in a network at time *t*, and |⋅| denotes the number of elements in a set.

For the WS model [31], we started from a circular regular network with eight degrees in each node. Next, we randomly rewired each edge with a probability *r*_*p*_ (0 ≤ *r*_*p*_ ≤ 1) so that the networks were strongly connected. This construction allowed us to adjust the network between the regular network (*r*_*p*_ = 0), small-world network (0 < *r*_*p*_ < 1), and the fully randomized network (*r*_*p*_ = 1).

The KE model [32] was constructed following a procedure similar to that for the BA model. In this model, each node had either an activated or a deactivated state, and we set a newly added node with *m* edges to an activated state. We started with a complete graph of *m* nodes with activated states. The *m* edges of the newly added node were connected to the following criteria; (i) *m* edges connect to the activated nodes, (ii) *m* edges connect to the existing nodes, including both activated and deactivated nodes using the preferential attachment whose probability was the same as that used to construct the BA model. The criterion (i) is selected with the probability *μ*(0 ≤ *μ* ≤ 1), and (ii) is selected with probability 1 − *μ* [32]. Next, one of the activated nodes was deactivated. The probability of choosing node *i* for deactivation is inversely proportional to its degree. In this study, the number of edges at each node for the regular networks (*r*_*p*_ = 0) of the WS model was set to 8, and *m* of the BA and KE models was set to 4.

Next, we normalized the node betweenness centrality, degree centrality, and the number of existing packets. To calculate the normalized node betweenness and normalized degree centralities, we first measured the node betweenness centrality of node *i*, *χ*_*i*_, and degree centrality of node *i*, *f*_*i*_. Those are defined as follows:

$$\begin{array}{c} \chi_{i}=\frac{\sum_{i_{s}=1;i_{s}\neq i}^{\left|V\right|}\;\sum_{i_{t}=1;i_{t}\neq i}^{i_{s}-1}\frac{g_{i}^{\left(i_{s},i_{t}\right)}}{N_{i_{s},i_{t}}}}{(|V|-1)(|V-2|)/2},\;\;\;i\in V, \end{array}$$ (3)

$$\begin{array}{c} f_{i}=\frac{k_{i}}{\sum_{j=1}^{\left|V\right|}k_{j}},\;\;\;i\in V, \end{array}$$ (4)

where $g_{i}^{\left(i_{s},i_{t}\right)}$ is the number of shortest paths from node *i*_*s*_ to *i*_*t*_ that pass through node *i*, $N_{i_{s},i_{t}}$ is the total number of shortest paths from node *i*_*s*_ to *i*_*t*_, and *k*_*i*_ is the degree of node *i*. The node betweenness centrality measures how often a node is on the shortest paths between any pairs of nodes in the networks, and the degree centrality measures the number of edges attached to the nodes. Next, we prepared vectors whose elements were the node betweenness and the degree centralities of nodes defined by $X={\left(\chi_{1},\ldots ,\chi_{\left|V\right|}\right)}^{⊺}$ and $F={\left(f_{1},\ldots ,f_{\left|V\right|}\right)}^{⊺}$, respectively. We then created a normalized vector by ***X***/||***X***|| and ***F***/||***F***||, where ‖ ⋅ ‖ denotes the Euclidean norm of a vector. To obtain normalized the number of existing packets, we counted the number of stored packets of node *i* during the simulation as $Q_{i}=\sum_{t=1}^{I}q_{i}\left(t\right)$, where *q*_*i*_(*t*) is the number of stored packets of the node *i* at the iteration *t*, and calculated the number of existing packet vectors as $Q={\left(Q_{1},\ldots ,Q_{\left|V\right|}\right)}^{⊺}$. ***Q*** was then normalized by ***Q***/||***Q***||.

**Fig 1:**
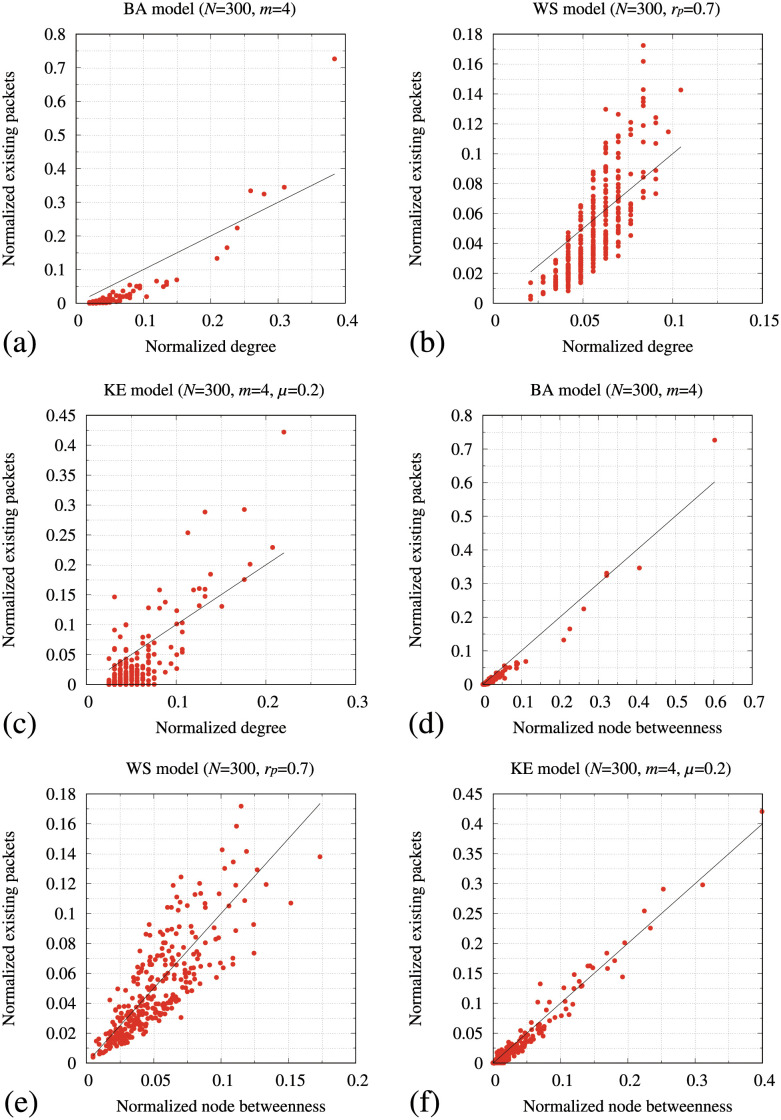
**Upper figures**: scatter plots of the normalized degree centrality of networks and normalized the number of existing packets generated by the SP method for the (a) BA, (b) WS, and (c) KE models. **Lower figures**: scatter plots of the normalized betweenness centrality of networks and normalized the number of existing packets generated by the SP method for the (d) BA, (e) WS, and (f) KE models. Black lines in all figures represent *f*(*x*) = *x*.

Fig 1 shows scatter plots between the normalized degree centrality and normalized the number of existing packets (upper figures) and scatter plots between the normalized node betweenness centrality and normalized the number of existing packets (lower figures) for the BA, WS, and KE models. Not surprisingly, in Fig 1(d)–1(f), the normalized node betweenness centrality and normalized the number of existing packets exhibit a stronger correlation than that of the normalized degree centrality (Fig 1(a)–1(c)), particularly in the KE model (Fig 1(c) and 1(f)). These results strongly suggest that *B*_*i*_ and *C*_*i*_ are defined based on the node betweenness centrality [27]. Thus, we modified the conventional packet transmission and storage performance of node *i*, expressed by ${\tilde{B}}_{i},{\tilde{C}}_{i},\forall i\in V$ as follows:

$$\begin{array}{c} {\tilde{B}}_{i}={\tilde{B}}_{\text{min}}+B_{\text{all}}{\tilde{b}}_{i}, \end{array}$$ (5)

$$\begin{array}{c} {\tilde{C}}_{i}={\tilde{C}}_{\text{min}}+\left\lfloor C_{\text{all}}{\tilde{b}}_{i}+0.5\right\rfloor , \end{array}$$ (6)

where ${\tilde{b}}_{i}$ is the normalized node betweenness centrality of node *i* defined by ${\tilde{b}}_{i}=b_{i}/\sum_{j=1}^{\left|V\right|}b_{j}$, and *b*_*i*_ is the node betweenness centrality of node *i*. *B*_all_ and *C*_all_ are control parameters that adjust $\sum_{i=1}^{\left|V\right|}B_{i}\approx \sum_{i=1}^{\left|V\right|}{\tilde{B}}_{i}$ and $\sum_{i=1}^{\left|V\right|}C_{i}\approx \sum_{i=1}^{\left|V\right|}{\tilde{C}}_{i}$ and are defined by $B_{\text{all}}=\sum_{i=1}^{\left|V\right|}\left(B_{i}-B_{\text{min}}\right)\geq 0$ and $C_{\text{all}}=\sum_{i=1}^{\left|V\right|}\left(C_{i}-C_{\text{min}}\right)\geq 0$, respectively. ${\tilde{B}}_{\text{min}}$ and ${\tilde{C}}_{\text{min}}$ guarantee that the node *i* has ${\tilde{B}}_{\text{min}}$ and ${\tilde{C}}_{\text{min}}$ at the minimum. We set *B*_min_ and *C*_min_ to 7 for all numerical simulations.

In sequence, we evaluated the transmission completion rate of packets for the conventional and improved communication network models. The transmission completion rate of packets is described as follows:

1. Transmission completion rate of packets *A*:
  $$\begin{array}{c} A=\frac{1}{RI}\sum_{t=1}^{I}a\left(t\right), \end{array}$$ (7)
  where *a*(*t*) is the number of packets that arrive at the destination at the *t*th iteration. If *A* is 1, all the packets are successfully transmitted to their destinations.

**Fig 2:**
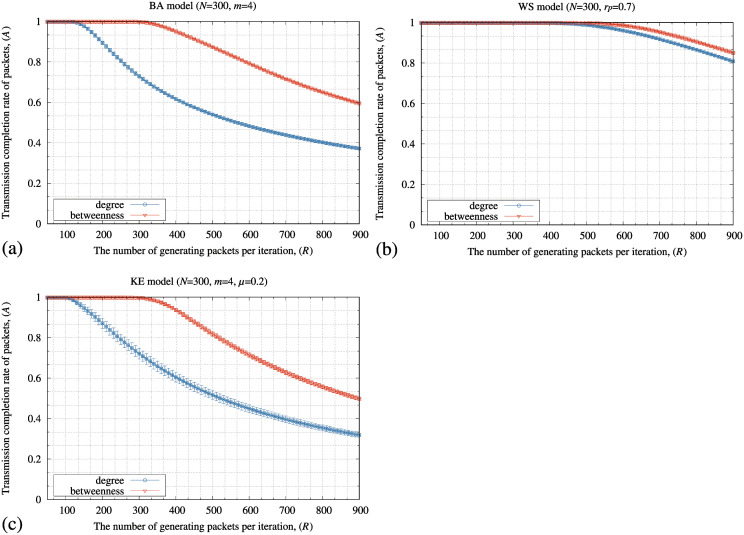
Relationship between the number of generating packets at each iteration (*R*) of the SP method and the transmission completion rate of packets (*A*) defined by Eq (7) for the (a) BA, (b) WS, and (c) KE models. In these figures, the “degree” and “betweenness” in the figure legends express that the packet transmission and storage performance are composed of the node degree and node betweenness centralities, respectively. In these figures, the standard deviation of each method is plotted as error bars.

Fig 2 shows the transmission completion rate of packets (*A*) defined by Eq (7) by the SP method for the conventional and improved transmission and storage performance of nodes. In these experiments, we constructed 30 different networks for every BA, WS, and KE model and averaged the results. These results indicated that the SP method maintained a higher *A* for the improved communication network in all the topologies of networks. From these results, we hereafter used the packet transmission and storage performance of nodes defined by Eqs (5) and (6) for later numerical experiments.

## Routing method using memory information

As our proposed routing method improves the conventional routing method using memory information [23, 24], we first describe this routing method and evaluate it for improved communication network models. To implement the routing method using memory information (hereafter referred to as the *memory* method), we first define the distance information, *ξ*_*ij*_, as follows:

$$\begin{array}{c} \xi_{ij}=\frac{d_{ij}+d_{jg(\pi_{i})}}{\sum_{s\in \partial_{i}}\left(d_{is}+d_{sg(\pi_{i})}\right)}=\frac{1+d_{jg(\pi_{i})}}{\sum_{s\in \partial_{i}}\left(1+d_{sg(\pi_{i})}\right)},\;\;\;\;i\in V,\;j\in \partial_{i}, \end{array}$$ (8)

where *d*_*ij*_ is the static shortest hop distance between the node *i* and its adjacent node *j*, *π*_*i*_ is a packet ready to be transmitted from node *i*, *g*(*π*_*i*_) is the destination node of *π*_*i*_, *d*_*jg*(*π*_*i*_)_ is the static shortest hop distance from the adjacent node *j* to *g*(*π*_*i*_), and ∂_*i*_ is the set of adjacent nodes of node *i*. The SP method is realized by using the distance information only; node *i* transmits a packet to its adjacent node *j* with the minimum value of *ξ*_*ij*_, namely ${\text{min}}_{j\in \partial_{i}}\xi_{ij}$.

Next, the memory information, *ζ*_*ij*,*τ*_, is defined as follows:

$$\begin{array}{c} \zeta_{ij,\tau }=\alpha \sum_{s=0}^{\tau }\gamma^{s}x_{ij}\left(\tau -s\right)=\alpha x_{ij}\left(\tau \right)+\gamma \zeta_{ij,\tau -1},\;\;\;\;i\in V,\;j\in \partial_{i},\;1\leq \tau \leq \omega_{ij} \end{array}$$ (9)

where *α* > 0 is a control parameter determining the strength of the memory information, *γ*(0 < *γ* ≤ 1) is a decay parameter of the memory information, *ω*_*ij*_ is the total number of transmissions of packets from node *i* to *j*, and *x*_*ij*_(*τ*) is a memorizing variable of transmission from node *i* to *j* at the *τ*th packet transmission defined as follows:

$$\begin{array}{c} x_{ij}\left(\tau \right)=\{\begin{array}{cc} 1 & \text{(if}\;\text{a}\;\text{node}\;i\;\text{transmits}\;\text{a}\;\text{packet}\;\text{to}\;\text{the}\;\text{node}\;j\;\text{at}\;\text{the}\;\tau \text{th}\;\text{packet}\;\text{transmission)},\\ 0 & \text{(otherwise)}. \end{array} \end{array}$$ (10)

Using the distance and memory information, we defined an evaluating function to determine the transmission of a packet from node *i* to its adjacent node *j* as follows:

$$\begin{array}{c} \underset{j\in \partial_{i}}{\text{min}}\;y_{ij,\tau },\;\;\;\;\;i\in V,\;1\leq \tau \leq \omega_{ij}, \end{array}$$ (11)

where *y*_*ij*,*τ*_ = *ξ*_*ij*_ + *ζ*_*ij*,*τ*_, and ∂_*i*_ ⊆ *V* is a set of adjacent nodes of node *i*.

In this method, the value of *ζ*_*ij*,*τ*_ increases if node *i* frequently transmits the packets to the adjacent node *j*. Therefore, node *i* avoids to transmit the next packet to the adjacent node *j* for a while because the smallest value of *y*_*ij*,*τ*_ in Eq (11) is determined as the next transmitting node. Thus, the memory information memorizes the past transmitting history at each node. Moreover, this method does not require additional information on the adjacent nodes, such as the number of stored packets, to diversify transmitting routes for packets.

We compared the transmission completion rate of packets by the memory, SP, *random SP* (SPr) methods, and the routing method by using the distance and packet stored distribution proposed in [2] for the networks generated from the BA, WS, and KE models. The SP method transmits a packet using uniquely determined shortest paths. In this work, we fixed indices of nodes after constructing a network. A node in the SP method transmits a packet to its adjacent node with the youngest index when multiple adjacent nodes satisfy $\underset{j\in \partial_{i}}{\text{min}}\;\xi_{ij}$. Moreover, a node in the SPr method randomly transmits a packet to one of its adjacent nodes with the smallest value of *ξ*_*ij*_. The routing method [2] determines the transmitting nodes of packets using the following equation:

$$\begin{array}{c} \Omega_{ij}\left(t\right)=\psi \left(d_{ij}+d_{jg(\pi_{i})}\right)+\left(1-\psi \right)q_{j}\left(t\right),\;i\in V,j\in \partial_{i}, \end{array}$$ (12)

where *q*_*j*_(*t*) is the number of stored packets in the adjacent node *j* at the *t*th iteration, and *ψ*(0 < *ψ* < 1) is a tunable parameter that determines the strength of the first and second terms. In the right-hand side of Eq (12), the first term is the shortest hop distance for the packet from node *i* to the destination of the packet through the adjacent node *j*, and the second term is the number of stored packets in the adjacent node *j*. In this method, the node *i* transmits a packet to its adjacent node *j* that has the smallest value of Ω_*ij*_(*t*). Our preliminary numerical experiments show that the performance of the routing method [2] is rapidly degraded if *R* becomes large because the values of ranges between the first and second terms are too different (see S1 Fig in S1 File for details); Eq (12) is dominated by the second term because $d_{ij}+d_{jg(\pi_{i})}\ll q_{j}(t)$ if *R* increases enough. To overcome this problem, we modified Eq (12) as follows:

$$\begin{array}{c} \Omega_{ij}^{\prime }\left(t\right)=\psi [\frac{d_{ij}+d_{jg(\pi_{i})}}{\sum_{s\in \partial_{i}}\left(d_{is}+d_{sg(\pi_{i})}\right)}]+\left(1-\psi \right)\frac{q_{j}\left(t\right)}{\sum_{s\in \partial_{i}}q_{s}\left(t\right)},\;i\in V,j\in \partial_{i}. \end{array}$$ (13)

We preliminarily conducted the numerical simulations to determine *ψ* for this routing method, and *ψ* was set to 0.9. Hereafter, we call the routing method using Eq (13) the *efficient routing*(*ER*) method.

In these numerical experiments, we set the number of iterations to *I* = 10^3^. In the memory method, we set *α* and *γ* in Eq (9) to 0.01 and 0.99 [24], respectively. We constructed 30 different networks for every BA, WS, and KE model and averaged the results.

**Fig 3:**
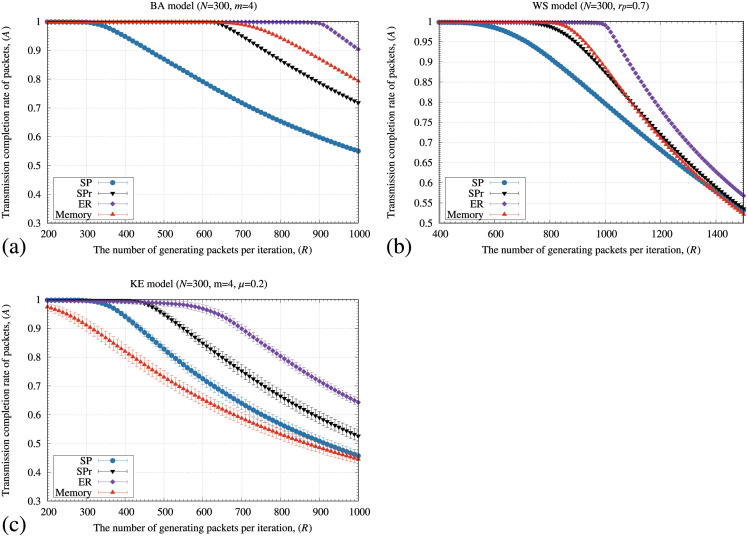
Relationship between the number of generating packets at each iteration (*R*) and transmission completion rate of packets (*A*) by the SP, SPr, ER, and memory methods for the (a) BA, (b) WS, and (c) KE models. In these figures, the standard deviation of each method is plotted as error bars.

Fig 3 illustrates the transmission completion rate of packets (*A*) by the SP, SPr, ER, and memory methods for the (a) BA, (b) WS, and (c) KE models as the number of generating packets (*R*) increased. In Fig 3(a) and 3(b), all the routing methods keep 100% of *A* if *R* is a small value. In addition, the value of *R* at which *A* decreased from 100% by the memory method is more significant than those of the SP and SPr methods for the BA and WS models. However, the memory method shows the lowest *A* in the KE model (Fig 3(c)). Although the memory method used the information of the transmitting node such as the distance and memory information, the ER method used information of both the transmitting and adjacent nodes such as the distance and the number of stored packets at adjacent nodes for each transmission of a packet. The ER method then retains the highest *A* value for all the network models. One of the reasons why the memory method shows poor performance for the KE model is triangular connections in the networks. For example, if nodes *a*, *b*, and *c* had a triangular connection, these nodes are mutually connected to each other. In this case, a packet starting from *a* is transmitted to *c* by using the following two paths; (i) the packet is directly transmitted to *c*, and (ii) the packet is first transmitted to *b* and next transmitted to *c*. The second path might be an unnecessary detour depending on the distribution of stored packets. The SP and SPr methods transmit the packets using the first path because these methods only consider the shortest distance. However, the memory method detours packets using the memory information obtained by the transmitting node. Thus, the packet might be unnecessarily detoured even if the packet should be transmitted directly to node *c* because of the unsuitable strength of the memory information. The performance of the memory method for the KE model was then degraded.

To clarify how many networks have triangular connections and how far apart are the nodes in the networks, we next measured the clustering coefficient ($\bar{\lambda }$) and the average shortest hop distance between nodes ($\bar{\kappa }$) [31]. The clustering coefficient ($\bar{\lambda }$) is defined as follows:

$$\begin{array}{ccc} \bar{\lambda } & = & \frac{1}{\left|V\right|}\sum_{i\in V}\lambda_{i}, \end{array}$$ (14)

$$\begin{array}{ccc} \lambda_{i} & = & \frac{2}{k_{i}\left(k_{i}-1\right)}\sum_{j\in V}\sum_{s\in V}a_{ij}a_{js}a_{si},\;i\in V, \end{array}$$ (15)

$$\begin{array}{c} a_{ij}=\{\begin{array}{cc} 1 & \text{(if}\;\text{node}\;i\;\text{and}\;j\;\text{are}\;\text{connected)},\\ 0 & \text{(otherwise)}. \end{array} \end{array}$$ (16)

In Eq (15), *a*_*ij*_*a*_*js*_*a*_*si*_ = 1 if the nodes *i*, *j*, and *s* are triangularly connected. Namely, Eq (15) averaged the number of triangular connections among the nodes *i*, *j*, and *s*. Therefore, the clustering coefficient ($\bar{\lambda }$) in Eq (14) measured an average ratio of triangular connections in the networks. If $\bar{\lambda }$ increases, the network has many triangular connections. The average shortest hop distance between nodes ($\bar{\kappa }$) is defined as follows:

$$\begin{array}{c} \bar{\kappa }=\frac{1}{\left|V|(|V|-1\right)}\sum_{i\in V}\sum_{j\in V,i\neq j}d_{ij}. \end{array}$$ (17)

If $\bar{\kappa }$ takes large values, the networks have long hop distances between nodes.

Table 1 shows the clustering coefficients ($\bar{\lambda }$) and the average shortest hop distance between nodes ($\bar{\kappa }$) averaged over 30 different networks generated by the BA, WS, and KE models. In Table 1, the KE model has the largest $\bar{\lambda }$ among all the models. In addition, the KE model has larger $\bar{\kappa }$ than those of the BA and WS models. From Fig 3 and Table 1, the memory method results in low *A* values for the networks with many triangular connections and long distances between nodes. If the network has large $\bar{\kappa }$, the packets need many hops to be transmitted to the destinations. Thus, the point where the value of *A* starts decreasing from 100% of the memory method for the KE model is lower than that for the SF and WS models because $\bar{\kappa }$ of the KE model is larger than the other models. Additionally, the performance of the memory method for the KE model is further degraded because of its triangular connections.

**Table 1:** $\bar{\lambda }$ and $\bar{\kappa }$ for the BA, WS, and KE models. In these simulations, we constructed 30 different networks for each model, and averaged the results.

	BA	WS (*r*_*p*_ = 0.7)	KE (*μ* = 0.2)
$\bar{\lambda }$	0.0941	0.038	0.491
$\bar{\kappa }$	2.741	2.373	3.423

## Routing method that adaptively adjusts the weight of memory information

From the results in previous section, the memory method showed a low transmission completion rate of packets for the KE model. To improve this method, we first investigate transmitting probabilities between any connected nodes. The transmitting probability of packets from node *i* and its adjacent node *j*, *p*_*ij*_(*I*), is defined as:

$$\begin{array}{c} p_{ij}\left(I\right)=\frac{\sum_{t=1}^{I}l_{ij}\left(t\right)}{\sum_{t=1}^{I}\sum_{s\in \partial_{i}}l_{is}\left(t\right)},\;\;\;\;i\in V,j\in \partial_{i}, \end{array}$$ (18)

where *l*_*ij*_(*t*) is the total number of packet transmissions from node *i* to its adjacent node *j* at iteration *t*.

**Fig 4:**
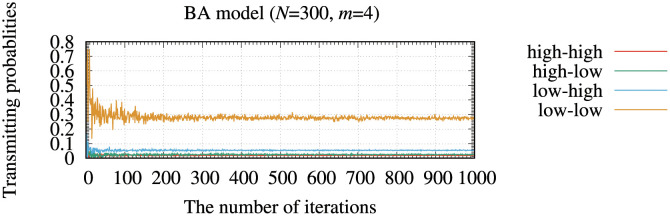
Typical examples of transmitting probabilities of four selected edges by the memory method for the BA model. In these simulations, we randomly selected four typical edges from different nodes in the networks. In this figure, “high-high,” “high-low,” “low-high,” and “low-low” are transmitting probabilities that connect high-degree nodes, high and low-degree nodes, low and high-degree nodes, and low-degree nodes, respectively.

Fig 4 illustrates the transmitting probabilities of the typical four edges by the memory method for the BA model. In these simulations, we randomly selected four typical edges from different nodes in the networks. Although transmitting probabilities converged to different values, each probability showed almost a constant value as the iterations progressed. These results lead to the following assumption: even if we incorporate the fixed transmitting probabilities into the memory information, instead of Eq (8), the routing method would show a similar performance as that of the memory method. To confirm this, we modified the memory information *ζ*_*ij*,*τ*_ by using the transmitting probabilities. The modified memory information, $\zeta_{ij,\tau }^{\prime }$, is defined as follows:

$$\begin{array}{c} \zeta_{ij,\tau }^{\prime }=\{\begin{array}{cc} \alpha \sum_{s=0}^{\tau }\gamma^{s}x_{ij}\left(\tau -s\right), & (t\leq L),\\ \frac{\left(k_{\text{max}}+k_{\text{min}}\right)}{2|V|}p_{ij}\left(L\right), & (L<t), \end{array} \end{array}$$ (19)

where *L* is a period of iterations for calculating the transmitting probability, and *k*_min_ and *k*_max_ are the minimum and maximum degrees in the networks, respectively. A coefficient (*k*_max_ + *k*_min_)/(2|*V*|) expresses a median of the degree and balances the weight of modified memory information. We heuristically found this weight in preliminary numerical simulations. In Eq (19), *p*_*ij*_(*L*), defined in Eq (18), is calculated when the iteration *t* is *L*. Further, we defined an evaluation function for this routing method as follows:

$$\begin{array}{c} \underset{j\in \partial_{i}}{\text{min}}\;y_{ij,\tau }^{\prime },\;\;\;\;i\in V,\;1\leq \tau \leq \omega_{ij}, \end{array}$$ (20)

where $y_{ij,\tau }^{\prime }=\xi_{ij}+\zeta_{ij,\tau }^{\prime }$.

Eqs (8), (10), (18), (19) and (20) compose the memory method with fixed transmitting probabilities (hereafter, we call this method *memory-pfix*. The memory-pfix method transmits the packets using the static paths determined by the distance and modified memory information. These paths might be better than the shortest paths by using the SP and SPr methods. We then compared the transmission completion rate of packets of the original memory method [24] with the memory-pfix method.

**Fig 5:**
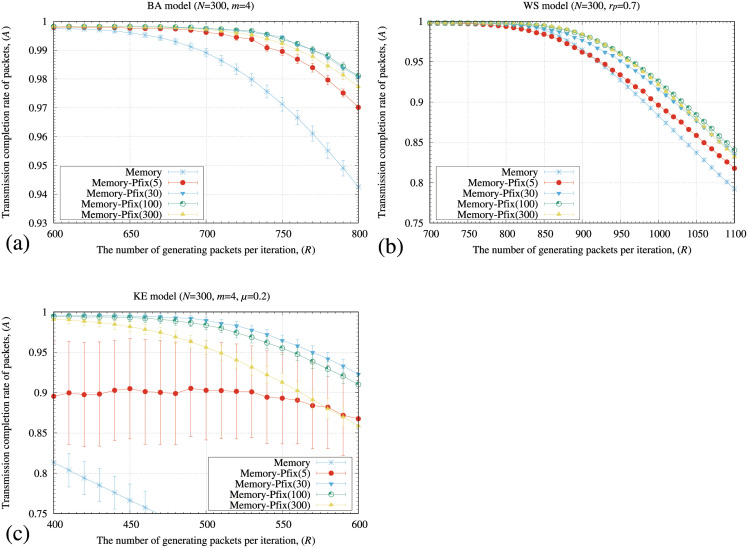
Relationship between the number of generating packets at each iteration (*R*) and a transmission completion rate of packets (*A*) by the original memory method and the memory-pfix method for the (a) BA, (b) WS, and (c) KE models. The numbers in the parentheses in the memory-pfix method indicate the periods of iterations for calculating the transmitting probability (*L*). In these figures, the standard deviation of each method is plotted as the error bars.

Fig 5 shows the transmission completion rate of packets (*A*) by the original memory and memory-pfix methods for the BA, WS, and KE models. We constructed 30 different networks generated by the BA, WS, and KE models and averaged the results. We used different periods of iterations for calculating the transmitting probability (*L*) and evaluated the method. The numbers in parentheses in the figure legends denote the *L* values. In Fig 5, the memory-pfix method showed higher *A* values compared to those of the original memory method for all the models.

**Fig 6:**
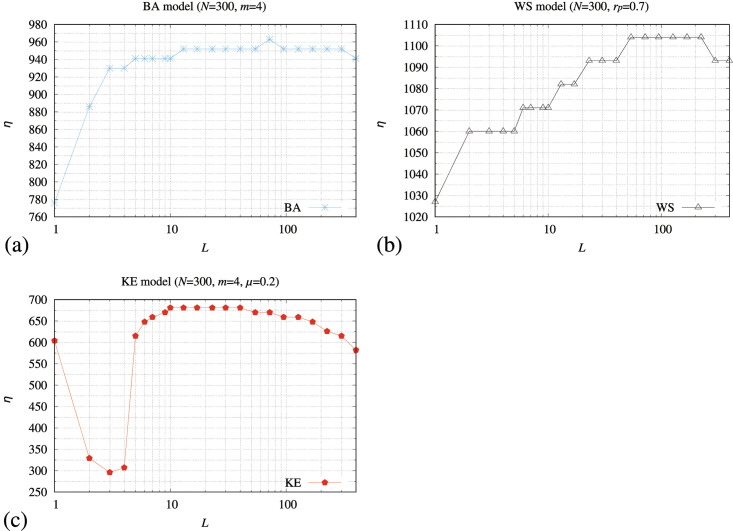
Relationship between a period of iterations for calculating the transmitting probability (*L*) in Eq (19) and the maximum value of *R*, where *A* in Eq (7) is lower than or equal to 0.9 of the memory-pfix method (*η*) for the (a) BA, (b) WS, and (c) KE models.

Fig 6 shows the maximum value of *R*, where *A* in Eq (7) is lower than or equal to 0.9 in the memory-pfix method (*η*) as a function of a period of iterations for calculating the transmitting probability (*L*) in Eq (19) for the BA, WS, and KE models. We constructed 30 networks for each model and calculated the averaged value of *η*. In these figures, the values of *η* are high when *L* > 10 for BA model (Fig 6(a)), 60 < *L* < 110 for WS model (Fig 6(b)), and 10 < *L* < 100 for KE model (Fig 6(c)).

One of the strategies to further enhance routing performance is to adjust *p*_*ij*_(*L*) depending on the states of adjacent nodes. To realize this, we changed $\zeta_{ij,\tau }^{\prime }$ in Eq (19) as follows:

$$\begin{array}{c} \phi_{ij,\tau }\left(t\right)=\{\begin{array}{cc} \alpha \sum_{s=0}^{\tau }\gamma^{s}x_{ij}\left(\tau -s\right), & (t\leq L),\\ {\tilde{p}}_{ij}\left(t\right), & (L<t), \end{array} \end{array}$$ (21)

where

$$\begin{array}{c} {\tilde{p}}_{ij}\left(t\right)=\frac{p_{ij}^{\prime }\left(t\right)}{\sum_{s\in \partial_{i}}p_{is}^{\prime }\left(t\right)}, \end{array}$$ (22)

$$\begin{array}{c} p_{ij}^{\prime }\left(t\right)=\{\begin{array}{cc} \frac{\left(k_{max}+k_{min}\right)}{2|V|}p_{ij}^{\prime }\left(t-1\right), & (\text{if}\;q_{j}\left(t\right)/{\tilde{C}}_{j}<\theta ),\\ \sqrt{\frac{k_{j}}{k_{max}}}, & (\text{otherwise}), \end{array} \end{array}$$ (23)

where we defined $p_{ij}^{\prime }\left(L\right)=p_{ij}\left(L\right)$. In Eq (23), *q*_*j*_(*t*) is the number of stored packets of node *j* at the *t*th iteration, ${\tilde{C}}_{j}$ is the modified transmission performance defined by Eq (6), and *θ* is a threshold value that determines whether the adjacent node *j* is congested. Eq (22) works to change the probability of transmitting packets to each of the adjacent nodes adaptively; when the number of stored packets in each adjacent node occupied over 100 × *θ*% of its buffer size, $p_{ij}^{\prime }\left(t\right)$ is recalculated by Eqs (22) and (23), thereby changing transmitting probabilities so that the packets can be transmitted to adjacent nodes with plenty of free space in their buffer after the iteration passes *L* in Eq (21). We defined that a node is in the free-flow state when $q_{j}\left(t\right)/{\tilde{C}}_{j}<\theta$ and in the congested state when $q_{j}\left(t\right)/{\tilde{C}}_{j}\geq \theta$. If the adjacent node is congested, the weight of memory information is set to $\sqrt{k_{j}/k_{max}}$.

In the networks, the nodes that transmit a large number of packets are divided into the following two types; (i) a node that has a small degree but has a large node betweenness centralities, and (ii) a node that has both a large degree and node betweenness centralities. We call the second type of node a *hub* node. If the first type of node is congested, the transmissions of packets to this node can be avoided by changing ${\tilde{p}}_{ij}\left(t\right)$ in Eq (23) to $\sqrt{k_{j}/k_{max}}$. However, our method continues to transmit the packets to the hub nodes even if these nodes are congested because if the hub nodes are congested, its ${\tilde{p}}_{ij}\left(t\right)$ becomes $\sqrt{k_{j}/k_{max}}≃1$, and this achieves $\{\left(k_{max}+k_{min}\right)/\left(2|V|)\right\}p_{ij}^{\prime }\left(t-1\right)<\sqrt{k_{j}/k_{max}}$. This implies that our method continues to use the hub node for transmitting packets independent of the amounts of packets in the networks because the hub nodes have high storage and transmission performance for routing the packets. In addition, our method tries to avoid the transmissions of the packets to the nodes that have small degree and large betweenness centralities. We expect that this strategy for avoiding the congestion of packets is effective for routing the packets in the networks.

Moreover, we redefined an evaluation function for our proposed method as follows:

$$\begin{array}{c} \underset{j\in \partial_{i}}{\text{min}}\;z_{ij,\tau }\left(t\right),\;\;\;\;i\in V,\;1\leq \tau \leq \omega_{ij}, \end{array}$$ (24)

where *z*_*ij*,*τ*_(*t*) = *ξ*_*ij*_ + *ϕ*_*ij*,*τ*_(*t*).

By using Eqs (8), (10), (21), (23) and (24), we finally propose a routing method that automatically adjusts the weight of memory information based on the memorized routing history. In our method, recalculations of distance information are not necessary once the networks are constructed. In addition, our method locally and less frequently exchanges information between the connected nodes; a node transmits a signal (0 or 1) to its adjacent nodes only if the node is congested (see S2 File for details). Hereafter, we call our proposed method *memory-pauto*.

Before starting the routing of packets, we prepared the routing tables at each node by calculating the shortest hop distance between any pairs of nodes. Because the SP method transmits the packets using the fixed transmitting paths, the method only refers to an index of the next adjacent node when routing the packets. The SP method then needs *O*(1) calculation cost at each node. However, the SPr method randomly selects an adjacent node if multiple adjacent nodes have the same shortest hop distance to the destination. If all the adjacent nodes of a transmitting node have the same shortest hop distances, and this transmitting node has the maximum degree, defined by *k*_max_, the SPr method needs *O*(*k*_max_) to determine the next transmitting node in the worst case. The ER method uses information of the shortest hop distances and the information of stored packets at adjacent nodes. This needs *O*(*k*_*i*_) at each node. Thus, the ER method requires *O*(*k*_max_) to determine the transmitting node in the worst case. The calculation costs by the memory-pauto method change depending on the values of *L* that determines the transmitting probabilities defined by Eq (21). When *t* < *L*, The memory-pauto uses the distance information in Eq (8) and modified memory information in Eq (21). At this time, each node needs *O*(1) to obtain the memory information between connected pairs of nodes because Eq (21) is the same as Eq (9). In addition, the calculation costs for obtaining the distance information are the same as the ones used in the ER method. Namely, each node in the memory-pauto method requires *O*(*k*_*i*_) when *t* < *L*. When *L* ≤ *t*, recalculations of transmitting probabilities by using Eqs (22) and (23) are required and these calculation costs become *O*(*k*_*i*_) at each node. Please note that this recalculation is required only if the state of the node changes. By summarizing these, in the worst case, the memory-pauto method requires *O*(*k*_max_) to determine the next transmitting node. When *t* = *L*, the memory-pauto calculates the transmitting probability of every edge by using Eq (22). This requires *O*(*Lk*_*i*_) at each node. However, this calculation only performed when *t* = *L*, and we omitted this from the calculation costs. All the routing methods require the distance information to transmit the packets. To obtain this information, we used the Dijkstra algorithm [30]. Generally, the Dijkstra algorithm needs *O*(|*V*|^2^), but it changes *O*(|*E*| + |*V*| log |*V*|) using a Fibonacci heap. Other methods such as the bread-first search or A* algorithm are also applicable to calculate the shortest distance between any pairs of nodes. However, these algorithms only performed when the networks were constructed. Therefore, we omitted this from the calculation costs for each routing method.

## Numerical experiments

The routing performance of the memory-pauto method mainly depends on *L* and *θ* in Eqs (21)–(23). *L* determines the period of iterations for calculating the transmitting probability, and *θ* determines whether the node is congested. We first evaluated the performance dependencies by these parameters.

**Fig 7:**
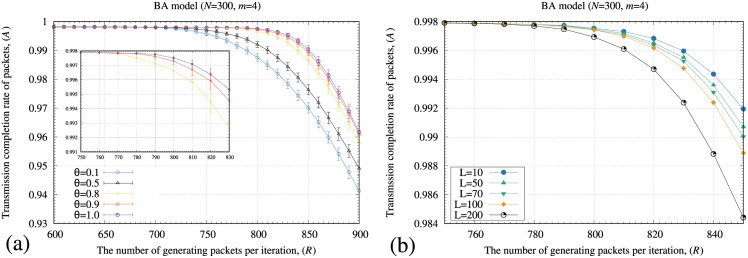
Relationships between the number of generating packets at each iteration (*R*) and a transmission completion rate of packets (*A*) for different *L* and *θ* values applied for the memory-pauto method. In these figures, the standard deviation of each method is plotted as error bars. In these numerical experiments, we used the BA model.

Fig 7 shows the transmission completion rate (*A*) by incorporating different *θ* and *L* values to the memory-pauto method. In Fig 7(a), the memory-pauto method maintains the highest *A* value if *θ* equals 1. In Fig 7(b), the memory-pauto method maintains the highest *A* value if *L* equals 10.

**Fig 8:**
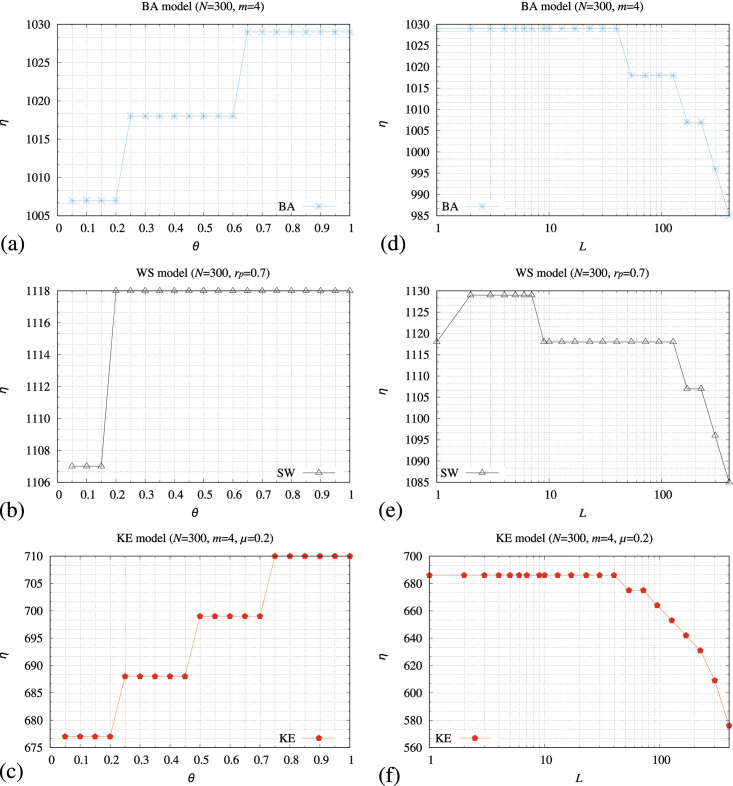
Relationships between a threshold (*θ*) that determines whether the adjacent nodes are congested and the maximum value of *R*, where *A* ≤ 0.9 (*η*) of the memory-pfix method for the (a) BA, (b) WS, and (c) KE models, and relationships between a period of iterations for calculating the transmitting probability (*L*) and *η* for the (d) BA, (e) WS, and (f) KE models. In these simulations, we set *L* to 5 in (a), (b), and (c), and *θ* = 1.0 in (d), (e), and (f).

Fig 8(a)–8(c) shows the maximum value of *R*, where *A* in Eq (7) is less than or equal to 0.9 of the memory-pfix method (*η*) when increasing the value of threshold (*θ*) that determines whether the adjacent nodes are congested. Fig 8(d)–8(f) shows the maximum value of *R*, where *A* in Eq (7) is less than or equal to 0.9 of the memory-pfix method (*η*) hen increasing the period of iterations for calculating the transmitting probability (*L*) in Eq (21). In these simulations, we set *L* to 5 in Fig 8(a)–8(c), and *θ* = 1.0 in Fig 8(d)–8(f). In Fig 8(a)–8(c), the memory-pauto shows the largest values of *η* if *θ* > 0.65 for BA, *θ* > 0.2 for WS, and *θ* > 0.75 for KE models. In Fig 8(d)–8(f), the memory-pauto shows the largest values of *η* when *L* ≤ 40 for BA, 2 ≤ *L* ≤ 7 for WS, and *L* ≤ 40 for KE models.

These results indicate that the memory-pauto method works well if each node holds as many packets as possible within a range of its modified transmission performance defined by Eq (6) because the method shows large *η* values if *θ* increases. Moreover, the memory-pauto method needs short periods to determine the transmitting probabilities because *η* takes large values if *L* decreases. Comparing with Fig 6, the memory-pauto method requires short periods for determining the transmitting probability because $p_{ij}^{\prime }\left(t\right)$ in Eq (23) changes depending on the states of adjacent nodes. Based on Fig 8, we set *θ* and *L* of the memory-pauto method to 1.0 and 5 for all the models, respectively.

We next evaluated the routing performance of the memory-pauto method for the BA, WS, and KE models. These numerical experiments evaluated the transmission completion rate of packets (*A*) in Eq (7). We also evaluated the average hop (*H*) and average arrival time (*U*) of arriving packets. The *H* and *U* are defined as follows:

1. Average hops of arriving packets *H*:
  $$\begin{array}{c} H=\frac{1}{\left|\nu \right|}\sum_{m\in \nu }h_{m}, \end{array}$$ (25)
  where *ν* is a set of arriving packets, |*ν*| is the number of elements in *ν*, and *h*_*m*_ is the number of hops from the source to the destination of packet *m*.
2. Average arrival time of arriving packets *U*:
  $$\begin{array}{c} U=\frac{1}{\left|\nu \right|}\sum_{m\in \nu }u_{m}, \end{array}$$ (26)
  where *u*_*m*_ is the number of iterations required to transmit packet *m* from the source to the destination.

In these simulations, we set *L* in Eq (18) for the memory-pfix to 70 for BA, 100 for WS, and 20 for KE models based on Fig 6. Other experimental conditions were the same as the ones described in previous sections. We constructed 30 different networks and averaged the results.

**Fig 9:**
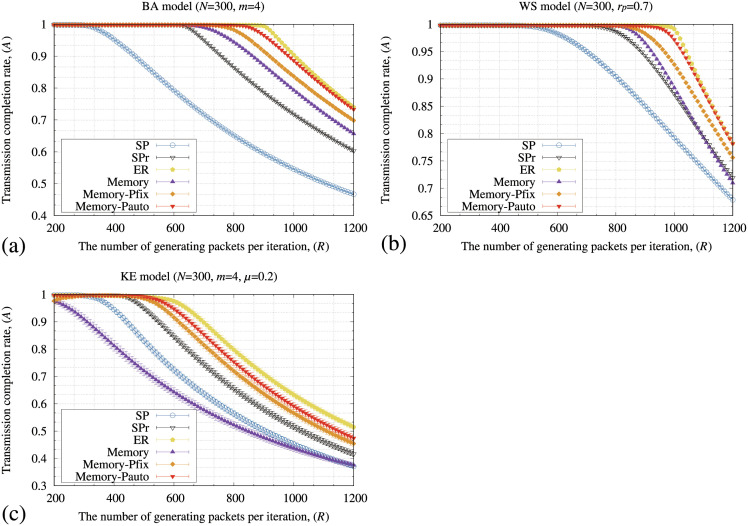
Relationships between the number of generating packets at each iteration (*R*) and a transmission completion rate of packets (*A*) of the SP, SPr, ER, memory, memory-pfix, and memory-pauto for the (a) BA, (b) WS, and (c) KE models. In these figures, the standard deviation of each method is plotted as error bars.

Fig 9 shows the transmission completion rate of packets (*A*) when the number of generating packets at each iteration (*R*) increases by the SP, SPr, ER, memory, memory-pfix, and memory-pauto methods for the BA, WS, and KE models. In Fig 9(a)–9(c), the memory-pauto method maintains a higher *A* than the SP, SPr, and memory-pfix methods for all the models. In particular, the maximum value of *R*, where *A* starts to decrease from 100%, is improved by approximately 170% by our proposed method for the BA model compared to the SP method and 25% compared to the memory method. In addition, the ER and memory-pauto methods show similar *A* values for the WS model. We also evaluated these routing methods for BA, WS and KE models with small degrees (see S3 Fig in S3 File for details).

**Fig 10:**
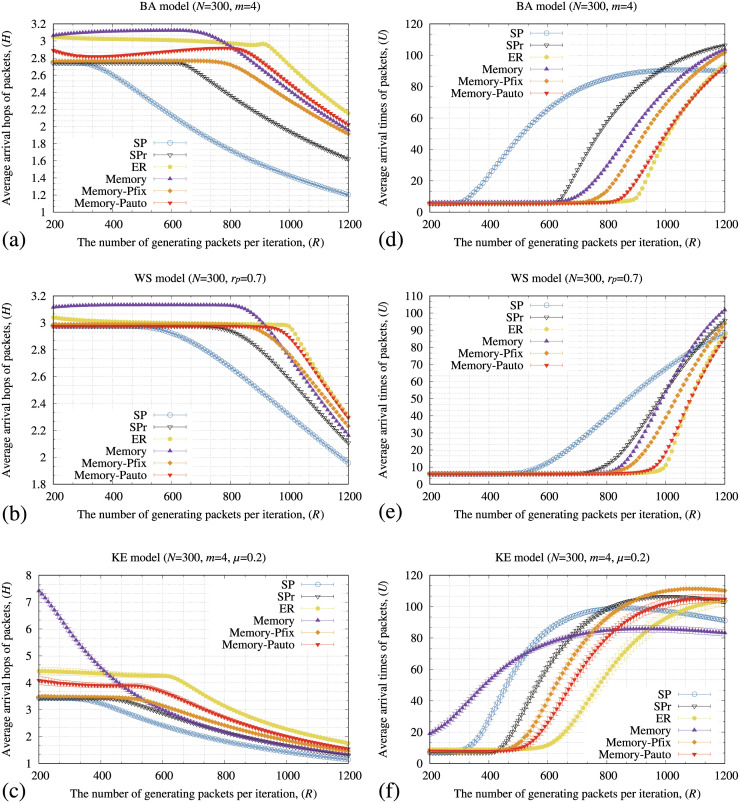
Relationships between the number of generating packets at each iteration (*R*) and the average hop of arriving packets (*H*) of the SP, SPr, ER, memory, memory-pfix, and memory-pauto methods for the (a) BA, (b) WS, and (c) KE models, and the average arrival times of arriving packets (*U*) for the (d) BA, (e) WS, and (f) KE models. In these figures, the standard deviation of each method is plotted as error bars.

Fig 10 shows the average arrival hops of arriving packets (*H*) and their average arrival time (*U*) by the SP, SPr, ER, memory, memory-pfix, and memory-pauto methods for the BA, WS, and KE models. In Fig 10(a)–10(c), the ER and memory-pauto methods show the longer *H* value as *R* increases compared to those for the the SP, SPr, memory, and memory-pfix methods. Moreover, the values of *H* for all the routing methods decreases as *R* increases because only the packets whose sources and destinations are directly connected arrive at their destinations when *R* increases enough. In Fig 10(d)–10(f), the memory-pauto exhibited long *H*, short *U*, and high *A* values. These results demonstrate that the memory-pauto method effectively diversifies the transmitting routes for packets, which increases the hops for the packets to the destinations.

Moreover, the total iterations of the packets to be transmitted from the sources to destinations are shortened by the effective transmissions. As a result, the proposed method shows the highest transmission completion rate even for large volumes of traffic flow in the communication networks.

In the above numerical experiments, we fixed the rewiring probability (*r*_*p*_) for the WS model to 0.7 and the probability (*μ*) that adjusts the clustering coefficient for the KE model to 0.2. However, the routing performance changes are based on the topologies of the networks. Thus, we clarified the performance dependencies against these probabilities.

**Fig 11:**
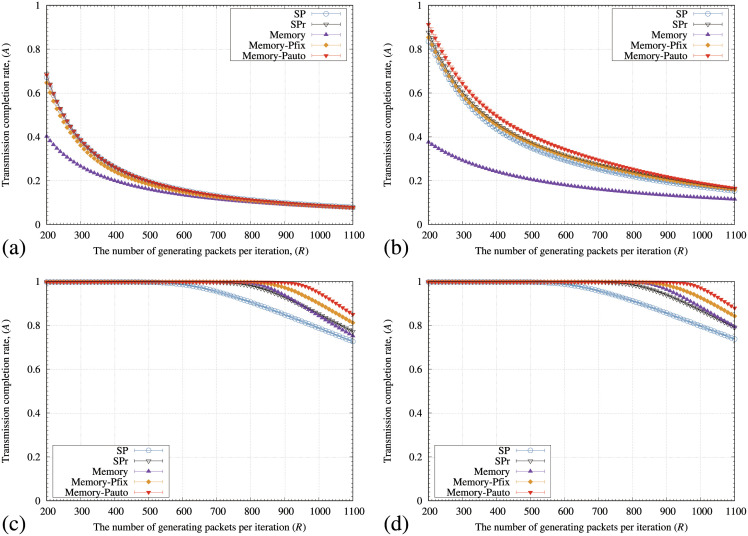
Relationships between the number of generating packets at each iteration (*R*) and the transmission completion rate of arriving packets (*A*) of the SP, SPr, memory-pfix, and memory-pauto methods for the WS model with (a) *r*_*p*_ = 0, (b) *r*_*p*_ = 0.01, (c) *r*_*p*_ = 0.5, and (d) *r*_*p*_ = 1.0. In these figures, the standard deviation of each method is plotted as error bars.

Fig 11 illustrates the transmission completion rate of packets (*A*) of SP, SPr, memory, memory-pfix, and memory-pauto methods for various values of the rewiring probability (*r*_*p*_). In Fig 11, the point where *A* starts to decrease from 100% by all the methods increases as *r*_*p*_ increases. In addition,*A* by the memory method is lower than those for the SP and SPr methods when the *r*_*p*_ values are 0 and 0.01. In contrast, the memory-pfix and memory-pauto methods maintain the higher *A* values than the SP and SPr methods for the WS model (Fig 11(c) and 11(d)).

**Fig 12:**
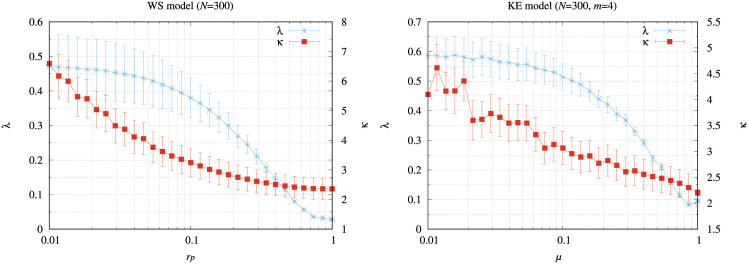
Relationships between the clustering coefficient ($\bar{\lambda }$) and the average shortest hop distance between nodes ($\bar{\kappa }$) if we change (a) the rewiring probability (*r*_*p*_) for the WS model, and (b) the probability (*μ*) that adjusts the clustering coefficient for the KE model. In these simulations, we constructed 30 different networks for each model, and averaged the results.

Fig 12 shows a relationship between the clustering coefficient ($\bar{\lambda }$) in Eq (14) and the average shortest hop distance between nodes ($\bar{\kappa }$) in Eq (17) if we changed the rewiring probability (*r*_*p*_) for the WS model (Fig 12(a)) and the probability (*μ*) that adjusts the clustering coefficient for the KE model (Fig 12(b)). In these simulations, we constructed 30 different networks for each model and averaged the results. In Fig 12(a), the WS model has large values of $\bar{\lambda }$ and $\bar{\kappa }$ when *r*_*p*_ is set to 0 and 0.01. In these cases, the networks have many triangular connections and long distances between the nodes. However, the WS model has the small values of $\bar{\lambda }$ and $\bar{\kappa }$ when *r*_*p*_ is set to 0.5 and 1. In these networks, the packets need a few hops transmitted to their destinations. In addition, the networks have a small number of triangular connections. The routing performance of all the methods is then improved as *r*_*p*_ increases (Fig 11(c) and 11(d)).

**Fig 13:**
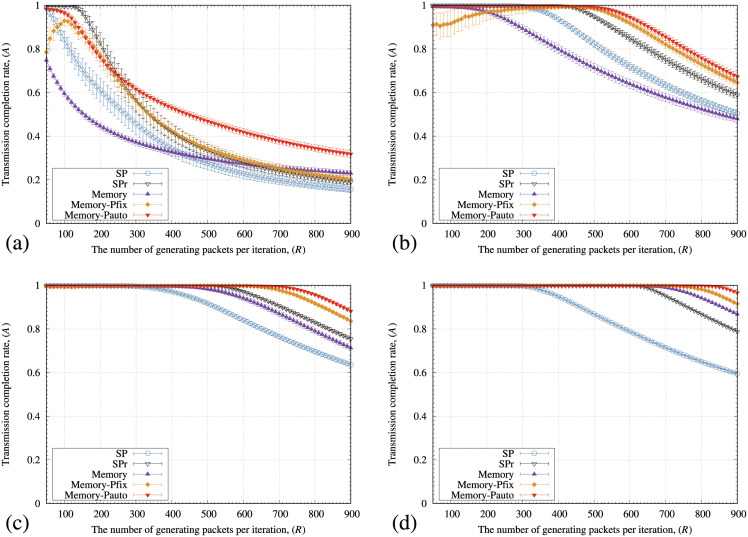
Relationships between the number of generating packets at each iteration (*R*) and a transmission completion rate of arriving packets (*A*) of the SP, SPr, memory, memory-pfix, and memory-pauto methods for the KE model with (a) *μ* = 0, (b) *μ* = 0.2, (c) *μ* = 0.5, and (d) *μ* = 1. In these figures, the standard deviation of each method is plotted as error bars.

Fig 13 illustrates the average arrival rate of packets (*A*) for various values of *μ* in the KE model. In Fig 13, the memory-pauto method shows the highest *A* value except for *μ* = 0. In addition, the maximum values of *R*, where *A* starts to decrease from 100% by the memory-pauto method, increased as *μ* increased. In Fig 12(b), the KE model shows small values of $\bar{\lambda }$ and $\bar{\kappa }$ as *μ* increases. In addition, the KE model changed to the SF model if *μ* approached 1. Because the triangular connections decrease and the networks have short distances when $\bar{\lambda }$ and $\bar{\kappa }$ have small values,*A* values for all the methods are improved (Fig 13(c) and 13(d)). Figs 11 and 13 indicate that the memory-pauto method showed high *A* values for the networks with many triangular connections and long distances between nodes.

These results indicate that the memory-pauto method effectively diversifies the routes of transmitting packets to maintain a high transmission completion rate by adjusting adequate weights of memory information at each node, depending on the traffic volumes and topologies of the communication networks.

## Conclusion

In this study, we improved the routing performance of the original routing strategy using memory information by automatically adjusting the weight of memory information based on states of adjacent nodes. First, we enhanced the routing performance of conventional communication network models by changing the packet transmission and storage performance based on their degree and on node betweenness centralities. Using the improved communication network models, the SP method, which is commonly applied in real communication networks was significantly improved. We also showed that the memory routing method maintained a high transmission completion rate compared to the SP method for the improved communication network models. However, the original memory routing method showed static transmitting probabilities as iteration moved, leading to the degradation of routing performance. Based on these consequences, we diversified the transmitting nodes of packets by changing weights of the memory information based on the transmitting probabilities between any connected nodes. Numerical simulations demonstrated that our proposed routing method improved transmission completion rate by approximately 170% compared to the SP method, and by 25% compared to the original routing method with the memory information for the communication networks with SF properties. Furthermore, our method displayed the highest transmission completion rate both for the networks with SF properties and also for those with highly clustered and with long distances between nodes. In future works, we plan to investigate the routing performance of our proposed method if the packet generating probability time-dependently changes depending on the local connectivity of networks.

## Supporting information

S1 File Performance evaluations of the original and modified ER methods. (PDF)

S2 File Performance evaluations with regard to the number of signals of the memory-pauto method. (PDF)

S3 File Performance evaluations of the network models with small and large degrees. (PDF)
