# Supplementary material for: A routing method with adaptively adjusting memory information based on local routing history
Source: PLoS One. 2023 Apr 19;18(4):e0283472. doi: 10.1371/journal.pone.0283472 (PMC10115277; doi:10.1371/journal.pone.0283472)
Supplement: S1 File — (PDF) [file pone.0283472.s001.pdf]

Supplementary Information: "A routing method with adaptively adjusting memory information based on local routing history"

Takayuki Kimura[1]* and Yutaka Shimada[2]

[1] *Faculty of Fundamental Engineering, Nippon Institute of Technology, 4-1-1 Gakuendai, Miyashiro, Saitama, 345-8501 Japan* and

[2] *Graduate School of Sciences and Engineering, Saitama University, 255 Shimo-Okubo, Sakura-ku, Saitama-shi, Saitama, 338-8570 Japan*

(Dated: March 18, 2023)

I. PERFORMANCE EVALUATIONS OF THE ORIGINAL AND MODIFIED ER METHODS

Fig. S1 shows the relationship between the number of generating packets at each iteration (R) and a transmission completion rate of packets (A) for the original and modified ER methods utilized by the BA, WS, and KE models. "*oER*" in the figure legend corresponds to the original routing method utilizing Eq. (12) [1], and "*ER*" corresponds to the modified routing method that uses Eq. (13). Numbers in the parentheses correspond to the values of ψ used in Eqs. (12) and (13). When $\psi = 1$, the oER and ER methods have a function equivalent to the SPr method. In these simulations, we set $I = 10^3$, $\beta = 10^3$, $\gamma = 0.4$, $B_{\min} = C_{\min} = 7$. We also set the number of edges attached to each node for the regular networks ($r_p = 0$) of the WS model to eight. In Fig. S1, the ER method excluding the cases wherein $\psi = 0.1$ and $\psi = 1$ retains larger A values than those retained by the oER method for the BA and WS models. In addition, the ER method with $\psi = 0.9$ retains the largest value of A if $R < 700$ among all the case of ψ for the KE method.

---

[1] P. Echenique, J. Gómez, and Y. Moreno, Europhysics Letters **71**, 325 (2005), ISSN 02955075.

*Electronic address: `tkimura@nit.ac.jp`

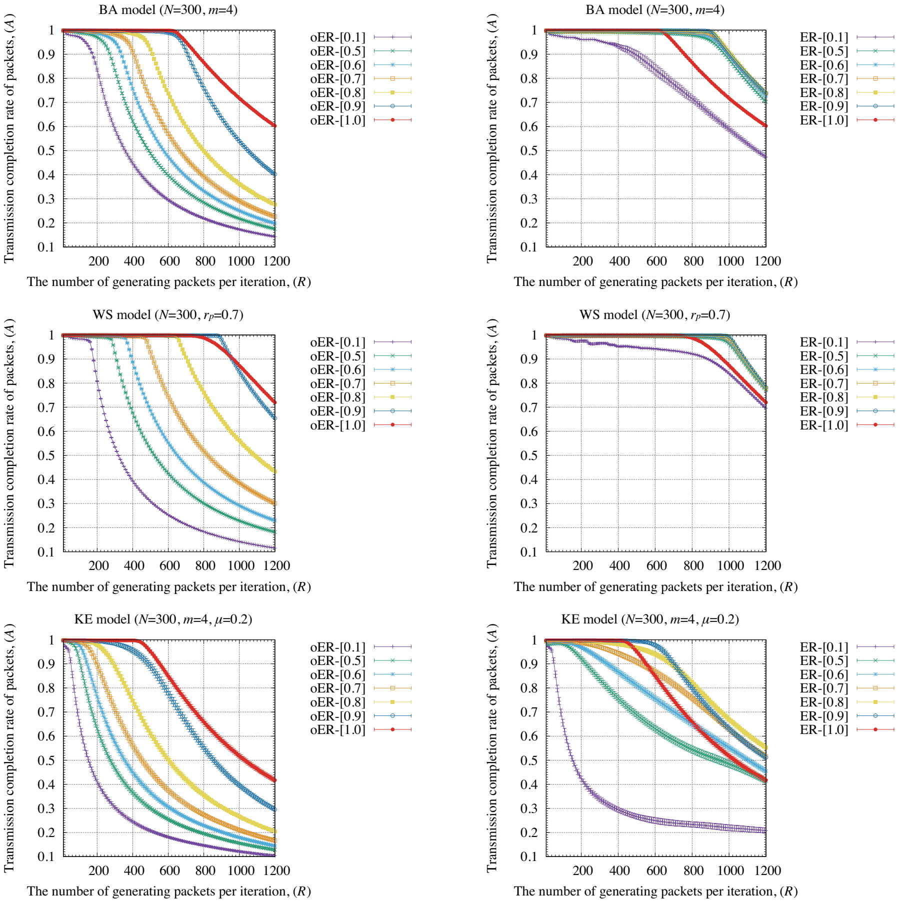

FIG. S1: Relationship between the number of generating packets at each iteration (R) and the transmission completion rate of packets (A) of the original and modified ER methods for the BA, WS, and KE models. In these figures, the standard deviation of each method is plotted as error bars. "oER" in the figure legend corresponds to the original routing method utilizing Eq. (12), and "ER" corresponds to the modified routing method utilizing Eq. (13). Numbers in the parentheses correspond to the values of ψ used in Eqs. (12) and (13).
