# Supplementary material for: A routing method with adaptively adjusting memory information based on local routing history
Source: PLoS One. 2023 Apr 19;18(4):e0283472. doi: 10.1371/journal.pone.0283472 (PMC10115277; doi:10.1371/journal.pone.0283472)
Supplement: S2 File — (PDF) [file pone.0283472.s002.pdf]

# Supplementary Information: "A routing method with adaptively adjusting memory information based on local routing history"

Takayuki Kimura[1*] and Yutaka Shimada[2]

[1] *Faculty of Fundamental Engineering, Nippon Institute of Technology, 4-1-1 Gakuendai, Miyashiro, Saitama, 345-8501 Japan and*

[2] *Graduate School of Sciences and Engineering, Saitama University, 255 Shimo-Okubo, Sakura-ku, Saitama-shi, Saitama, 338-8570 Japan*

(Dated: March 18, 2023)

## II. PERFORMANCE EVALUATIONS WITH REGARD TO THE NUMBER OF SIGNALS OF THE MEMORY-PAUTO METHOD

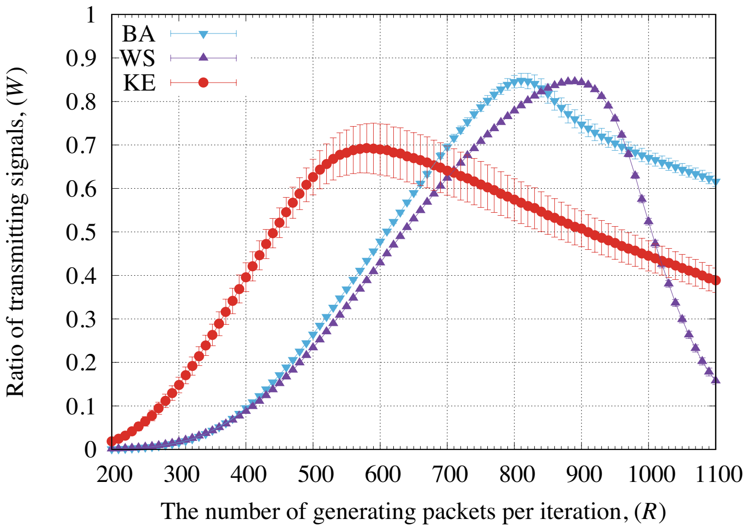


FIG. S2: Relationships between the number of generating packets at each iteration ($R$) and the ratio of signals ($S$) transmitted using the memory-pauto method for the BA, WS, and KE models. In these figures, the standard deviation of each method is plotted as error bars.

Fig. S2 shows a relationship between the number of generating packets at each iteration ($R$)

*Electronic address: `tkimura@nit.ac.jp`

and the ratio of the number of signals ($W$) transmitted using the memory-pauto method for the BA, WS, and KE models. In these simulations, we set the values of the parameters to the same ones depicted in Fig. 9 of the main text. We define the ratio of transmitted signals ($W$) as follows:

$$W = \frac{1}{\sum_{i \in V} k_i \lceil \frac{P_i}{\tilde{C}_i} \rceil} \sum_{i \in V} \sum_{t=1}^{I} w_i^{(t)}, \tag{S1}$$

$$w_i^{(t)} = \begin{cases} k_i & \text{(if the state of node } i \text{ changes from free-flow to congested and vice versa at iteration } t\text{)}, \\ 0 & \text{(otherwise)}, \end{cases} \tag{S2}$$

where $P_i$ is a set of packets transmitted from node $i$, $k_i$ is the degree of node $i$, $I$ is the total number of iterations, and $w_i^{(t)}$ is the number of signals transmitted by node $i$ at iteration $t$ using the memory-pauto method. We define a node to be in the free-flow state when $q_j(t)/\tilde{C}_j < \theta$ and in the congested state when $q_j(t)/\tilde{C}_j \geq \theta$ (see Eqs. (21)–(23) for detail). The ER method always considers the number of stored packets at adjacent nodes for the packet transmission. Thus, the node $i$ in the case of the ER method requires $k_i$ signals to recognize the number of stored packets at adjacent nodes for transmission of each packet. Therefore, node $i$ requires its adjacent nodes to transmit the information regarding the number of stored packets when node $i$ transmits the packets; if node $i$ transmits $P_i$ packets in total during the simulations, the adjacent nodes of node $i$ will need to transmit $\lceil \frac{P_i}{\tilde{C}_i} \rceil$ signals, and thus, the total number of signals transmitted over the networks is $\sum_{i \in V} k_i \lceil \frac{P_i}{\tilde{C}_i} \rceil$ when using the ER method, where $\lceil \cdot \rceil$ is the ceiling function and $\tilde{C}_i$ is the modified transmission performance (see Eq. (6) in the main text for detail). However, each node in the memory-pauto method transmits signals to its adjacent nodes only when the ratio of the number of stored packets ($q_i(t)$) to the transmission performance ($\tilde{C}_i$) exceeds threshold $\theta$ (see also Eqs. (21)-(23) in the main text). Therefore, when $q_i(t)/\tilde{C}_i$ exceeds $\theta$ or $q_i(t)/\tilde{C}_i$ is below $\theta$, node $i$ transmits $k_i$ signals to inform the adjacent nodes of its state changes. The total number of signals transmitted from node $i$ in the memory-pauto method during the simulations is therefore $\sum_{t=1}^{I} w_i^{(t)}$. In other words, the ratio of transmitted signals ($W$) indicates the number of signals transmitted by the memory-pauto method in comparison with that transmitted using the ER method.

In Fig. S2, it is observed that all the values of $W$ are less than 0.9. This indicates that using the memory-pauto method, the small number of signals can be transmitted, at most less than 10%, to the adjacent nodes.
