# Supplementary material for: A routing method with adaptively adjusting memory information based on local routing history
Source: PLoS One. 2023 Apr 19;18(4):e0283472. doi: 10.1371/journal.pone.0283472 (PMC10115277; doi:10.1371/journal.pone.0283472)
Supplement: S3 File — (PDF) [file pone.0283472.s003.pdf]

Supplementary Information: "A routing method with adaptively adjusting memory information based on local routing history"

Takayuki Kimura[1*] and Yutaka Shimada[2]

[1] *Faculty of Fundamental Engineering, Nippon Institute of Technology, 4-1-1 Gakuendai, Miyashiro, Saitama, 345-8501 Japan and*

[2] *Graduate School of Sciences and Engineering, Saitama University, 255 Shimo-Okubo, Sakura-ku, Saitama-shi, Saitama, 338-8570 Japan*

(Dated: March 18, 2023)

III. PERFORMANCE EVALUATIONS OF THE NETWORK MODELS WITH SMALL AND LARGE DEGREES

Fig. S3 shows the relationship between the number of generating packets at each iteration (R) and the transmission completion rate of the packets (A) of the SP, SPr, ER, memory, memory-pfix, and memory-pauto methods for the BA, WS, and KE models. In Fig. S3(a), (b), and (c), we set the value of m for the BA and KE models to two, and the number of edges attached to each node in the regular networks (υ) of the WS model to four. In Fig. S3(d), (e), and (f), we set the value of m and υ to four and eight, respectively. These subfigures (Fig. S3(d), (e), and (f)) are the same as the ones depicted in Fig. 9. In these simulations, we set the parameters excluding the number of edges for each model to the same values used depicted in Fig. 9 of the main text. In Fig. S3(a), (b), and (c), it can be observed that the values of R tend to decrease more significantly with the decrease in the value of A for all the models as compared to the models with large degrees (Fig. S3(d), (e), and (f)). Interestingly, the memory-pauto method retains a higher value of A than the ER method for the BA model which has small degrees (Fig. S3(a) and (d)). Because models with small degrees have half the number of edges than those with large degrees, there are also fewer transmitting routes between the connected nodes.This results in the performance degradation of the routing methods.

*Electronic address: `tkimura@nit.ac.jp`

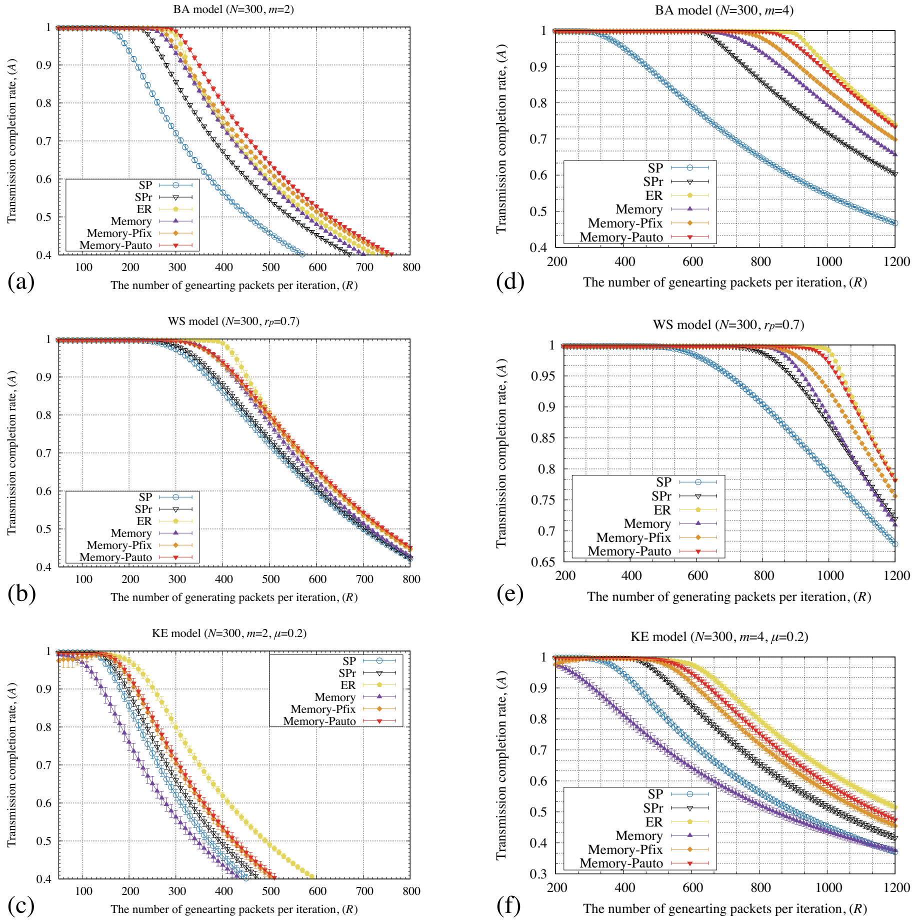

FIG. S3: Relationship between the number of generating packets at each iteration (R) and the transmission completion rate of the packets (A) for the SP, SPr, ER, memory, memory-pfix, and memory-pauto methods utilized by the BA, WS, and KE models. In (a), (b), and (c), we set the value of m for the BA and KE models to two, and the number of edges attached to each node in the regular networks (v) of the WS to four. In (d), (e), and (f), we set the value of m and v to four and eight, respectively. In the figures, the standard deviation of each method is plotted as error bars.
